# Deciphering the Potentials of Cardamom in Cancer Prevention and Therapy: From Kitchen to Clinic

**DOI:** 10.3390/biom14091166

**Published:** 2024-09-18

**Authors:** Shabana Bano, Avisek Majumder, Ayush Srivastava, Kasturi Bala Nayak

**Affiliations:** 1 Department of Medicine, University of California, San Francisco, CA 94158, USA; 2 Department of Neurological Surgery, University of California, San Francisco, CA 94158, USA; 3 Quantitative Biosciences Institute, Department of Medicine, University of California, San Francisco, CA 94158, USA

**Keywords:** cancer prevention, chemoprotectant, chemoprevention, *Elettaria cardamomum*, ayurveda, inflammation, antioxidants, anti-microbial, diet, cancer therapy, epigenetics, natural medicine, pharmacological use, traditional Indian medicine, phytochemicals, flavonoids

## Abstract

Cardamom (cardamum) is a spice produced from the seeds of several Elettaria and Amomum plants of the Zingiberaceae family. Cardamom has been demonstrated to offer numerous benefits, including its antioxidant, antimicrobial, anti-inflammatory, and other metabolic (anti-diabetic) properties, and its potential to reduce cancer risk. Recently, researchers have extracted and tested multiple phytochemicals from cardamom to assess their potential effectiveness against various types of human malignancy. These studies have indicated that cardamom can help overcome drug resistance to standard chemotherapy and protect against chemotherapy-induced toxicity due to its scavenging properties. Furthermore, chemical compounds in cardamom, including limonene, cymene, pinene, linalool, borneol, cardamonin, indole-3-carbinol, and diindolylmethane, primarily target the programmed cell death lignin-1 gene, which is more prevalent in cancer cells than in healthy cells. This review provides the medicinal properties and pharmacological uses of cardamom, its cellular effects, and potential therapeutic uses in cancer prevention and treatment, as well as its use in reducing drug resistance and improving the overall health of cancer patients. Based on previous preclinical studies, cardamom shows significant potential as an anti-cancer agent, but further exploration for clinical use is warranted due to its diverse mechanisms of action.

## 1. Introduction

Cancer is the second leading cause of death worldwide, and in 2022, 1,918,030 new cancer cases and 609,360 cancer deaths were estimated in the United States [1]. Although there has been a massive development in the treatment of cancer through conventional therapies, drug resistance and drug-associated cytotoxicity make it even more challenging [2,3]. In the realm of cancer research, the exploration of complementary and alternative medicine has piqued the interest of scientists [4,5]. Notably, Ayurveda, the ancient Indian system of natural medicine centered around plant-based remedies, holds significant promise in preventing and treating various severe ailments, including cancer [6,7,8]. These achievements present an exciting opportunity for further exploration and collaboration between traditional Ayurvedic practice and modern medical research for cancer treatment. In Ayurvedic medicine, many herbs have already been proven under clinical studies to help prevent, heal, and reduce side effects and complications associated with cancer [9].

**Figure 1:**
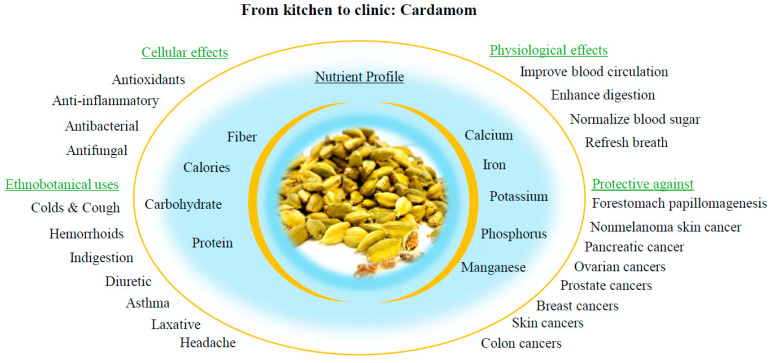
Versatile effects of cardamom in health and disease.

According to the World Health Organization (WHO), 80% of the global population uses plant-based remedies for a healthy lifestyle [10]. Additionally, historical records show that civilizations dating back to 2600 BC used various plants for medicinal purposes [11]. It is fascinating that Indian Ayurvedic medicines include 516 plant-based drugs that have the potential to combat cancer, fight microbes, reduce inflammation, and boost the immune system [12]. Their effects depend on the bioactive compounds (known as Flavonoids) present in these medicinal plants. Flavonoids have diverse structures and offer potential health benefits for fighting cancer [13]. Different flavonoids in cardamom have shown medicinal properties, including reducing inflammation, acting as an antioxidant, and maintaining cellular homeostasis, as illustrated in Figure 1 [14]. Interestingly, sources for several of these plant-based drugs are already part of everyday cooking. One such ingredient is cardamom, which has a rich nutrient profile and contains several flavonoids that give it medicinal properties such as anti-inflammatory, antioxidant, and maintenance of cellular homeostasis, as illustrated in Figure 1 [14].

Recently, a significant focus has been on exploring naturally occurring compounds that can actively combat cancer by inhibiting or slowing down its development. It is evident that dietary spices (commonly found in our kitchen) hold great promise for developing innovative strategies in cancer management. When used in appropriate concentrations, spices and their phytochemicals have demonstrated a powerful potential to regulate various cancer-related processes. As we discussed in this paper, cardamom contains cancer-fighting compounds that show promise in effectively eradicating cancer cells. We used the term “kitchen to Clinic” in the title to highlight the potential of a commonly found kitchen ingredient as a medicinal product [15]. Nonetheless, further comprehensive investigation through preclinical and clinical studies is essential to support the recommendation of this spice for cancer prevention and treatment. This review delves into the medicinal properties and therapeutic uses of cardamom, detailing its cellular effects and potential applications in cancer prevention and treatment. It also discusses cardamom’s role in combating drug resistance and improving the overall health of cancer patients.

## 2. Different Cardamom Products, Their Extractions, Handling, and Storage

Cardamom contains many compounds like essential oils, terpenes, phenolic acids, and flavonoids [16]. Different essential oils present in cardamom include 1,8-cineole, α-terpineol, and limonene [16]. Cardamom’s aromatic properties are credited to these oils, which also give its anti-inflammatory and antimicrobial effects [17]. Cardamom contains terpenes like sabinene and myrcene, which have antioxidant and anti-inflammatory properties [18]. On the other hand, phenolic acids present in cardamom include compounds such as caffeic acid and ferulic acid, which have been found to contribute to its antioxidant and anti-inflammatory effects [16]. Moreover, cardamom contains various flavonoids, including quercetin, kaempferol, and luteolin. These compounds are known for their antioxidant, anti-inflammatory, and anti-cancer properties [16]. The flavonoid content in cardamom is generally measured in micrograms per gram of dried seeds. For instance, quercetin content in cardamom seeds has been reported to be around 3.24 mg per gram of dried seeds. Hence the specific amounts of flavonoid found in cardamom can vary depending on the cardamom variety and processing methods.

Various extraction procedures have been used to obtain different phytochemicals found in cardamom [19,20]. These extraction methods have been classified as conventional (e.g., hydro distillation, steam distillation, and Soxhlet extraction) and advanced extraction methods (e.g., enzyme-assisted, instant controlled pressure drop, microwave-assisted, pressurized liquid, solar energy based, subcritical, supercritical fluid, and ultrasound-assisted extractions), resulting in a highly concentrated product that must be stored in dark, airtight containers to maintain its stability. In order to extend the shelf life of cardamom seed extract, it should be handled carefully to prevent contamination and stored in cool, dark conditions. In order to purify flavonoids and terpenes, chromatography must be used to isolate the compounds, and sealed containers must be kept away from light and moisture [21]. To preserve the quality and efficacy of cardamom ground powder, they are typically stored in airtight containers away from heat and moisture. Keeping the bioactive components of cardamom products intact and ensuring their therapeutic potential requires precise handling and storage.

## 3. Overview of Cardamom in Health and Disease

**Figure 2:**
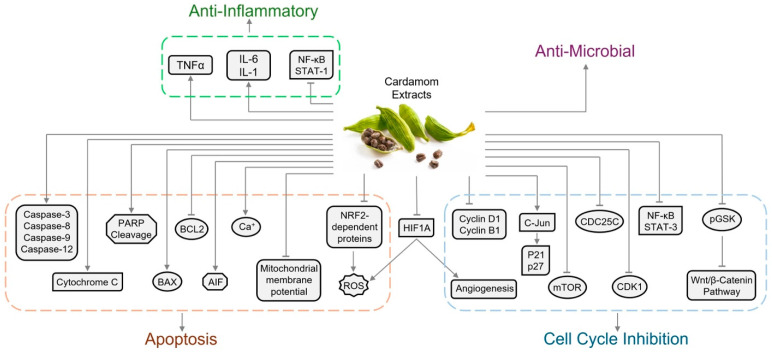
Molecular targets of cardamom extracts and its cellular effects. Abbreviations: AIF (apoptosis inducing factor), BCL2 (B-cell lymphoma 2), BAX (Bcl-2-associated X protein), HIF1A (Hypoxia-inducible factor-1), IL-1 (interleukin-1), NF-κB (nuclear factor kappa-light-chain-enhancer of activated B cells transcription factor), NRF2 (nuclear factor erythroid 2-related factor 2), PARP (poly ADP-ribose polymerase), pGSK (phospho-Glycogen synthase kinase), ROS (reactive oxygen species), STAT-1 (signal transducer and activator of transcription 1), TNFα (tumor necrosis factor alpha).

Food is a fundamental necessity for human survival [22]. Herbs and spices are intrinsically connected to food, with extensive data highlighting their health benefits. Hippocrates stated, “Let food be thy medicine and medicine be thy food” [23]. Spices, primarily described as aromatic plants, enhance food flavor and provide nutritional value [24,25]. Historically, spices have been used as condiments due to their aroma, taste, and color. They also have a long history in folk medicine for treating various diseases in the presence of various bioactive compounds [26]. Among spices, cardamom (scientific name: *Elettaria cardamomum* (L.) Maton) is a significant member of the Zingiberaceae family and the Elettaria Maton genus plant [27]. It is also known as “chhoti elaichi” or “true cardamom” in India, and holds the title of “Queen of Spices”. The cardamom seed extract is a concentrated liquid derived from the seeds of the cardamom [28]. It is a key ingredient in polyherbal formulations for treating Alzheimer’s disease-related dementia [29]. It is also used in herbal combinations for treating anxiety, tension, and insomnia [30]. An in vivo study on an ayurvedic formulation containing cardamom demonstrated CNS-depressant and anticonvulsant activities in mice [31]. In traditional Indian medicine, cardamom is used as a digestive aid and for treating flatulence [32]. Due to its soothing properties, it is also incorporated into massage oils, lotions, soaps, detergents, and perfumes [33]. Different Ethnobotanical uses of cardamom are summarized in Table 1 and illustrated in Figure 2.

An in vitro study on estradiol permeation through hairless mouse skin indicated that complex terpenes in cardamom enhance the transdermal permeation of moderately lipophilic drugs like estradiol [34]. Furthermore, in vivo and in vitro studies on indomethacin permeation revealed significant enhancement after pre-treatment with cardamom oil, attributed to the presence of cyclic monoterpenes from *Elettaria cardamomum* [35]. Diindolylmethane (DIM) and indole-3-carbinol (I3C) are beneficial natural compounds found in cardamom known to inhibit cancer-associated processes and regulate hormone balance in breast cancer [36]. The bioactive compounds in cardamom, such as limonene, caffeic acid, and cineole, downregulate several signaling pathways in cancer cells by inhibiting cyclooxygenase-2 and cytochrome P450 [37]. In the subsequent paragraphs, we summarized the medical properties and therapeutic application of cardamom, along with its potential impact on cancer prevention, treatment, and the reduction of drug resistance in cancer patients.

**Table 1:** Ethnobotanical Uses of *Elettaria cardamomum*.

Ethnobotanical Uses as	References
As an abortifacient, diuretic, stomachic, laxative, and carminative; in asthma, hemorrhoids, bronchitis, scabies, strangury, pruritis; diseases of the bladder, kidney, rectum, and throat; earache, snake bite, inflammation, headache, and scorpion sting	[38]
CNS-depressant and anticonvulsant activities	[31]
Treating sore throat, asthma, colds, coughs, bladder and kidney diseases, burning sensations, flatulence, scanty urine, heart weakness, indigestion, and piles	[39]
As an anti-flatulent and to stimulate the appetite	[40]
Neutralize poisons, reduce Kapha (one of the three doshas in Ayurveda), enhance skin complexion, and alleviate itching	[41]

## 4. Medicinal Properties and Pharmacological Uses of Cardamom

Cardamom is renowned for its medicinal properties, particularly its efficacy in treating stomach disorders. Using preclinical models, researchers demonstrated the gastroprotective effects of cardamom. Cardamom is also beneficial for digestive issues, including nausea, vomiting, and indigestion (Table 2). The presence of numerous bioactive compounds in cardamom imparts a pleasant aroma, and chewing the seeds can help alleviate bad breath. Additionally, chewing cardamom seeds provides a cooling effect, which is beneficial for treating sore throat, asthma, colds, coughs, bladder and kidney diseases, burning sensations, flatulence, scanty urine, heart weakness, indigestion, and piles [39].

Cardamom is also utilized as a carminative, digestive aid, expectorant, purgative, stimulant, thirst reliever, deodorant, diuretic, and tonic. Historically, it has been noted for its ability to neutralize poisons, reduce Kapha (one of the three doshas in Ayurveda), enhance skin complexion, and alleviate itching [41]. Table 2 provides further details on the uses of cardamom.

**Table 2:** Medicinal Properties of *Elettaria cardamomum* and pharmacological uses of cardamom.

Activities	Tested Using	References
Anti-inflammatory	Cardamom oil	[42]
The antidote to snake venom	Cardamom crude extract	[41]
Gastroprotective	Cardamom oil and crude extract	[43]
Inhibitor of Human Platelet Aggregation	Cardamom crude extract	[44]
Anti-bacterial	Cardamom seed extract and oil	[45,46]
Inhibition of viral inhibition		[47]
Anti-cancer	Cardamom crude extract	[48]
Antioxidant	Cardamom extracts and oil	[45,46,49]
Insecticidal activity	Cardamom oil	[35]
Analgesic	Cardamom oil	[50]
Anti-fungal	Cardamom oil	[51]
Anti-diabetic	Cardamom crude extract	[52]
Post-Operative Nausea and Vomiting	Cardamom oil	[53]

## 5. The Bioactive Compounds of Cardamom and Their Signaling Targets

Kaempferol, a primary phytoconstituent of cardamom, is a natural dietary flavonol belonging to the flavonoid class [54]. Luteolin, another commonly found flavonoid, is present in various plants, such as vegetables and medicinal herbs, with a particularly high concentration in spices [55]. Specific targets of luteolin are listed in Table 3. Kaempferol is also involved in several signaling pathways, some of which are detailed in Table 3. Additionally, other bioactive compounds, including quercetin, resveratrol, and eucalyptol, along with their target molecules, are detailed in Table 3.

## 6. Anti-Cancer Activity of Cardamom

Cancer is a process of irregular cell growth and proliferation caused by genetic and environmental factors [71,72]. Cancer cells are often found to utilize more nutrients, micronutrients, and other compounds to sustain high energy requirements and cellular turnover [71,73,74,75,76]. Cancer cells have been found to use more metabolites through methionine and folate cycles than normal cells [77,78]. Methionine is a precursor of the methionine cycle, which is converted to S-Adenosyl methionine (SAM), and that is the only methyl group donor for epigenetic alteration of DNA, RNA, and histone [79,80]. Research suggests that a high methionine diet may contribute to the development of different types of cancer by causing epigenetic alterations in the genome [5,55,65,66,67,68]. This indicates a potential interaction between genetic predisposition and environmental factors in the onset of cancer [5,71,81,82,83,84]. In this regard, changes in dietary patterns and the utilization of plant-based compounds hold a solid base for preventing and treating cancer [85]. Numerous natural products, including herbs, spices, pulses, and nuts, have undergone extensive testing against cancer cell lines in preclinical and clinical phases. The successful isolation of anti-cancer drugs such as docetaxel, paclitaxel, and topotecan from plants and herbs highlights the immense potential of these bioresources [86]. These natural sources contain bioactive compounds that have demonstrated promising therapeutic properties against human cancer cell lines. Notably, cardamom has shown the ability to inhibit proliferation, migration, and invasion while also inducing apoptosis through the involvement of Wnt/β-catenin, NF-κB, and PI3K/Akt pathways [87]. Further studies are needed to comprehensively understand the biosafety and pharmacokinetic profile of cardamom, in order to establish its potential as a viable candidate for cancer treatment.

Cardamom, a dietary phytoproduct, demonstrates significant potential as a natural cancer treatment due to its bioactive compounds [88]. Numerous animal studies have confirmed its anti-cancer properties. Bhattacharjee and Rana demonstrated its efficacy in inhibiting colon carcinogenesis in Swiss albino mice [89]. Further studies suggest that cardamom and its active components exhibit protective effects against experimentally induced colon cancer [16]. Research by Qiblawi et al. indicated the chemopreventive effects of cardamom on chemically induced skin carcinogenesis in mice [90]. Additionally, in another preclinical trial, Qiblawi et al. confirmed the chemopreventive effects of cardamom against Benzo(α)Pyrene-induced forestomach papillomagenesis in Swiss albino mice [91].

Phytochemicals in cardamom, such as cineole and limonene, have shown protective roles against cancer progression. Vutakuri and Somara (2018) highlighted the use of *Elettaria cardamomum* (L.) Maton in natural and herbal medicine for breast cancer [92]. Among its bioactive compounds, kaempferol stands out due to its remarkable pharmaceutical activity [93]. Kaempferol, a dietary flavonoid, plays a significant role in human health and cancer chemoprevention [93]. Lee, Hwang, and Choi (2016) reported that kaempferol inhibits pancreatic cancer growth by blocking the EGFR-related pathway in vitro [94]. Further details on the significant bioactive compounds in cardamom and their effects on different types of cancer are provided in Table 4.

## 7. Cellular Effects of Cardamom in the Prevention and Treatment of Cancer

The development of cancer can be influenced by both genetic and environmental factors. Factors such as diet and lifestyle play a significant role in increasing the risk of cancer [106,107]. Many traditional spices are already noted for their anti-cancer properties, so adding these ingredients to our daily routine reduces the risk of cancer to a certain extent [108]. Cardamom has antioxidative, anti-inflammatory, anti-proliferative, and antimicrobial properties, which may complement standard cancer treatments. Although prevention is ideal, it is not always possible, and conventional treatments such as surgery, radiation, and chemotherapy often cause significant systemic complications. It appears that cardamom, along with other herbs and spices, could enhance the effectiveness or alleviate the side effects of established cancer treatments, rather than serve as a complete substitute [108].

Cardamom, a versatile spice used in a variety of culinary and medicinal applications, has gained increasing attention for its potential role in cancer prevention and treatment. The area of chemoprevention, which involves using natural or synthetic agents to prevent, inhibit, or reverse cancer development, has become an increasingly important research area in recent years as scientists seek alternative approaches to battling the disease [109]. The chemo-preventive agent cardamonin has been extensively studied for a variety of cancers, including those of the breast, hematological system, gastrointestinal tract, and colon [110].

Cardamonin, an active analog of chalcone, has shown promising results; specifically, it inhibits the human triple-negative breast cancer cell invasiveness by downregulation of Wnt/β-catenin signaling cascades and reversal of epithelial-mesenchymal transition [111,112]. Additionally, it inhibits apoptosis and cycle arrest via signaling pathways like the notch receptor, Wnt/b-catenin, and PI3K/AKT/mTOR, which are crucial for cancer progression and development [113,114]. A xenograft mouse model with co-administered cardamonin and doxorubicin inhibited tumor growth and CSC accumulation, further supporting the potential of this spice in chemoprevention [96]. Other epidemiological studies suggest populations with a high cardamom consumption may have a lower risk of certain types of cancer, reinforcing the potential of this spice in chemoprevention [115]. The development of cardamom-based nutraceuticals and functional foods could provide another natural and accessible means to harness cardamom’s chemoprevention properties [14]. In the following sections, we discuss the molecular targets of cardamon and its cellular effects through its anti-oxidative, anti-inflammatory, anti-proliferative, and anti-microbial properties.

### 7.1. Anti-Oxidative Effects of Cardamom

Antioxidants are molecules that combat free radicals, thereby protecting cells from harmful effects [116]. Cardamom is recognized for its antioxidant properties due to the presence of various bioactive compounds [117]. It contains flavonoids such as kaempferol, quercetin, luteolin, and pelargonidin, which are responsible for their antioxidant activity, as shown in Table 5 [118,119]. Further studies have confirmed the antioxidant profiles of quercetin, kaempferol, and isoquercitrin [120]. Cardamom also includes 1,8-cineole, a compound with significant antioxidant capability to neutralize free radicals [121]. The antioxidant activity of quercetin was corroborated by Zheng et al. in 2016 [26]. Additionally, Winarsi et al. (2016) demonstrated that an ethanolic extract of cardamom rhizome enhances the antioxidant and immune status in atherosclerotic rats subjected to adrenaline and egg yolks [52]. The reaction mechanisms of these bioactive molecules remain primarily unexplored, necessitating further investigation to elucidate their mechanisms of action.

### 7.2. Anti-Inflammatory Activity of Cardamom

Cardamom is recognized for its anti-inflammatory properties, attributed to bioactive compounds such as quercetin, kaempferol, and isoquercitrin [124]. Numerous animal studies have confirmed the potent anti-inflammatory activity of cardamom. For instance, a study by Sermugapandian et al. demonstrated that cardamom oil (which contains bioactive compounds including α-terpinyl acetate, 1,8-cineole (eucalyptol), limonene, and terpinene) exhibits anti-inflammatory effects on carrageenan-induced paw edema in rats by modulating serum levels of tumor necrosis factor α, interleukin 6, and interleukin 1 [125]. Additionally, quercetin and kaempferol have shown significant anti-inflammatory effects by targeting different pathways, such as inhibiting NF-κB activation and STAT-1 [126].

### 7.3. Anti-Microbial Effects of Cardamom

Cardamom extracts exhibit varying degrees of anti-microbial activity against various microorganisms [127]. Cardamom has demonstrated anti-bacterial effects against several bacterial species, including Enteropathogenic Escherichia coli (EPEC), a pathotype of *Escherichia coli* responsible for causing diarrhea in young children [128]. Additionally, Listeria monocytogenes, a Gram-positive, rod-shaped bacterium causing listeriosis through contaminated food, is susceptible to cardamom extract [129]. Another Gram-positive, rod-shaped bacterium, *Bacillus pumilus,* has also shown sensitivity to cardamom extracts [130].

Studies have revealed the anti-microbial activity of both whole plants and plant products of cardamom, as shown in Table 6. Jebur et al. (2014) demonstrated that extracts from cardamom fruit and leaf oil exhibit anti-microbial activity against various bacteria, including *Streptococcus pneumoniae*, *Pseudomonas aeruginosa*, *Enterobacter* spp., *Klebsiella pneumoniae*, *Staphylococcus epidermidis*, *Streptococcus pneumoniae*, *Staphylococcus aureus*, *Proteus mirabilis*, *Escherichia coli*, *Serratia* spp., and *Salmonella typhi* [131]. An assay conducted by Agaoglu et al. (2005) showed that cardamom seeds possess inhibitory activity against *Klebsiella pneumonia*, *Candida albicans*, *Staphylococcus aureus*, *Micrococcus luteus*, *Enterococcus faecalis*, and *Mycobacterium smegmatis* [132]. Recent studies have attributed the anti-microbial activity of cardamom to the presence of various chemical compounds, including volatile oils, alkaloids, phenols, tannins, and lipids [45,131].

### 7.4. Metabolic Effects of Cardamom

Many systemic disorders like obesity, diabetes, and hyperhomocysteinemia have been linked to cancer [138,139,140,141,142]. Reversing the above metabolic conditions may have significant consequences for reducing the risk of cancer. Common features of the above-mentioned metabolic conditions are oxidative stress, ER stress, and inflammation [75,143,144,145,146]. Cardamom has been found to reduce oxidative stress and inflammation (as discussed above), possibly reducing cancer risk. Nevertheless, more research is needed to confirm these effects.

Cardamom, like other botanical supplements, appears to possess anti-diabetic properties and may help manage metabolic syndromes that can lead to heart disease and type 2 diabetes [147]. Cardamom has been studied in vivo and in vitro for its hypoglycemic and anti-diabetic properties. In an animal study that fed rats high carbohydrate and fat (HCHF) diets, the rodents that were also given cardamom powder lost weight and had healthier cholesterol than those not fed this supplement. According to the results of this study, cardamom supplementation reduced cholesterol levels increased by the HCHF diet [148].

In a clinical trial, pre-diabetic women who were overweight or obese took 3 g of cardamom daily for two months. Results revealed a decrease in total cholesterol from 192.6 to 183.7 mg/dL, a decrease in bad cholesterol (LDL) from 118.1 to 110.5 mg/dL, and a slight decrease in good cholesterol (HDL) from 44.1 to 42.7 mg/dL [149]. In another study, 3 g of cardamom per day for 10 weeks was administered as a supplement significantly decreased triglycerides (TG) in 83 T2DM patients from 158.4 to 125.8 mg/dL when compared with a placebo [150].

A supercritical carbon dioxide extraction method was used to obtain high-purity extracts from cardamom, and it has also been found to increase insulin sensitivity in the liver and glucose uptake in the gut [151]. The extract of this spice showed potential benefits for treating type 2 diabetes, suggesting that it may complement traditional treatments such as metformin and BGR-34. However, further research is needed to confirm its efficacy and therapeutic potential as a treatment option [152].

Cardamom supplements can lower cholesterol, decrease triglycerides, reduce inflammation and oxidative stress markers, and improve insulin sensitivity and glucose uptake [139,142,153]. In addition to showing potential as an alternative to insulin treatment for people with type 2 diabetes, it warrants further clinical trials.

### 7.5. Cardamom as a Chemoprotectant

Chemotherapy can lead to significant toxicity in other tissues, adversely affecting the patient’s quality of life. To mitigate these effects without compromising the primary drug’s efficacy, chemoprotectants, such as synthetic molecules like amifostine, aprepitant, dexrazoxane, filgrastim, sargramostim, mesna, oprelvekin, palifermin, and recombinant human erythropoietin have been developed [154,155]. In natural compounds, many flavonoids and polyphenols have been reported to work as chemoprotectants [156,157,158,159,160,161]. However, no studies have been conducted on cardamom as an effective alternative for chemoprotection due to its antioxidant and anti-inflammatory properties.

### 7.6. Cardamom for Reducing Chemotherapeutic Drug Resistance

Overcoming chemotherapeutic drug resistance is a critical goal in improving cancer treatment. Recent studies have shown that various cancer cell lines, when exposed to clinically relevant concentrations of 5-fluorouracil, doxorubicin, or paclitaxel, exhibited an elevated expression of IL-6, IL-8, and MCP-1 cytokines [96]. Furthermore, it was also noticed that the activation of NF-κB and Stat3 pathways was up-regulated. Studies have indicated that IL-6 and IL-8 play a crucial role in breast tumorigenesis, are associated with poor patient survival, and are implicated in chemotherapy resistance [doi: 10.18632/oncotarget.5819: 5,49–51,99]. Moreover, the NF-κB and Stat3 pathways govern cytokine production in response to diverse stimuli, are associated with drug resistance, and regulate tumor angiogenesis and invasiveness [16,43,44,49,52,53,54,96]. Different studies (as mentioned in Section 7.2) suggest that cardamom effectively represses the up-regulation of IL-6, IL-8, and MCP-1, as well as the activation of NF-κB and Stat3. These effects underscore the potential of cardamom to inhibit resistance to chemotherapeutic drugs.

## 8. Study the Therapeutic Effects of Cardamom in Different Cancer Types

### 8.1. Effects of Cardamom on Breast Cancer

Breast cancer is the second most common cause of cancer-related deaths in women globally [162,163]. Studies have demonstrated the potential beneficial effects of cardamom against triple-negative breast cancer [36]. Khairani et al. showed that Amomum cardamomum seeds display anti-cancer activity against breast cancer by preventing growth and inducing apoptosis [97]. Kong et al. demonstrated that treating MDA-MB231 and MCF-7 cells with cardamonin led to the halting of cell division in the G2/M phase by reducing the levels of cyclin D1, and also induced cell death [164]. Moreover, cardamonin found in cardamom was also found to inhibit the growth of breast cancer cells by reducing the activity of the Wnt/β-catenin pathway by inhibiting phosphorylated glycogen synthase kinases (GSKs) [111]. Research conducted by Jin et al. on MDA-MB-231 breast cancer cells demonstrated the ability of cardamonin to facilitate programmed cell death induced by reactive oxygen species (ROS) through HIF-1a signaling [165]. In another study, cardamonin suppressed the growth of drug-resistant breast cancer stem cells and stopped these stem cells from renewing themselves by inhibiting the NF-κB and STAT3 pathways [96]. In this study, cardamom has shown promising results when combined with chemotherapy drugs by lowering pro-inflammatory factors IL-6 and IL-8 [37]. Reports have shown that cardamonin reduced the movement and invasion of triple-negative breast cancer cells (TNBC, BT-549 cells) by reversing the process of epithelial-mesenchymal transition (EMT) [166,167].

### 8.2. Effects of Cardamom on Cervical Cancer

Addressing the significant issue of the high mortality rate from cervical cancer among women in low- and middle-income groups due to lack of awareness and affordable treatment is critical [168]. A study demonstrated the potential of cardamom treatment for attenuating cell proliferation in mTOR inhibitor-resistant HeLa cervical cancer cells by reducing the phosphorylation of mTOR and S6K1, indicating a promising avenue for further research and development [169].

### 8.3. Effects of Cardamom on Colon Cancer

Colon cancer is the third most common cancer in the United States, affecting both men and women. Although the death rate has decreased over the past decade, it is still expected to lead to around 55,000 deaths in 2020 [170]. Bhattacharjee et al. revealed that aqueous suspensions of cinnamon and cardamom enhanced the level of detoxifying enzyme (GST activity) with a simultaneous decrease in lipid peroxidation during chemically induced colon carcinogenesis in Swiss albino mice [89]. Some promising research has shown that cardamonin, when combined with other chemotherapeutic agents like 5-FU, can effectively reduce the growth of chemo-resistant colon cancer cells and induce apoptosis [171]. Cardamonin has also been found to activate caspase-3 and caspase-9, increase the expression of pro-apoptotic Bax, decrease the expression of anti-apoptotic Bcl-2, and regulate various factors involved in cell cycle arrest [171]. Additionally, it downregulates the expression of testes-specific protease 50 and NF-κB p65 [171]. In a separate study, cardamonin has been shown to inhibit β-catenin response transcription in colon cancer cells, induce G2/M cell cycle arrest, reduce levels of β-catenin, inhibit cyclin D1 and c-Myc, and inhibit β-catenin/TCF-4, thus demonstrating therapeutic potential in cells with APC gene mutations without affecting mRNA levels [98].

### 8.4. Effects of Cardamom on Gastric Cancer

Gastric cancer is the fifth most commonly diagnosed cancer and the third deadliest globally, with a survival rate of less than 5% [172,173]. Research shows that cardamonin is a promising treatment for AGS cells, effectively inhibiting cell proliferation and migration, and promoting apoptosis [174]. This compound downregulates the expression of the anti-apoptotic Bcl-2 protein while increasing Bax and caspase-3 levels [174]. In a controlled clinical trial, it was shown that cardamom has a chemopreventive effect against benzo(α)pyrene-induced forestomach papillomagenesis in Swiss albino mice. Moreover, cardamonin found in cardamom suppresses the expression of key markers associated with cancer progression, such as Snail, Slug, and Vimentin, while increasing E-cadherin expression levels [175]. This study highlighted the synergistic effect of cardamonin when combined with 5-FU, leading to enhanced chemosensitivity of BCG-823 through the inhibition of the Wnt/β-catenin pathway [175]. The above two studies demonstrate the potential of cardamonin to exert an anti-cancer effect, either alone or in combination with other drugs.

### 8.5. Effects of Cardamom on Glioblastoma Cancer

Glioblastoma is a highly aggressive type of brain cancer that commonly affects adults and is known for its resistance to chemotherapy and its metastatic nature [176]. Cardamonin has demonstrated the ability to increase the sensitivity of CD133 + GSCs [177]. This is achieved by inhibiting Bcl-2, Bcl-xL, and Mcl-1 proteins, along with increased Bax levels, reducing cell proliferation and enhancing apoptosis. Furthermore, cardamonin decreases levels of survivin and VEGF by docking in nuclear STAT3 activation, with high binding energies of −10.78 kcal/mol, indicating a highly energetic exothermic process [177,178].

### 8.6. Effects of Cardamom on Leukemia

Cardamonin has exhibited promising anti-leukemic activity against leukemia cells. According to a study by Liao et al., cardamonin decreased cell viability and promoted apoptosis in WEHI-3 mice cells in vitro by influencing various proteins [179]. This study revealed that cardamonin increased ROS activity and the production of Ca2+, leading to ROS-induced apoptosis. Moreover, it downregulated mitochondrial membrane potential (MMP), while upregulating caspase-3, caspase-8, and caspase-9 [179]. An increase in pro-apoptotic factors, such as cytochrome C, AIF, Bax, Endo G release, and caspase-12, was observed, alongside a decrease in the anti-apoptotic Bcl-2 after cardamonin pretreatment [179].

### 8.7. Effects of Cardamom on Lung Cancer

Lung cancer is a significant contributor to cancer-related deaths globally [180]. Studies have shown that cardamonin exhibits promising anti-cancer effects against lung cancer in laboratory settings. In non-small-cell lung cancer (NSCLC) cell experiments, cardamonin triggered apoptosis by activating caspase-3, increasing Bax levels, and decreasing Bcl-2 levels [181]. It also induced cell cycle arrest in the G2/M phase by reducing cyclin D1/CDK4 levels [181]. Additionally, cardamonin reversed the epithelial-mesenchymal transition (EMT) process and regulated downstream factors such as PI3K and mTOR signaling, thereby suppressing the invasion and migration of NSCLC cells [181]. Similarly, another study demonstrated that cardamonin reduced the proliferative, invasive, and migratory properties of LLC cells by targeting Snail and other downstream targets, including ribosomal S6K1, and inhibiting NF-κB [99]. In a separate study, two cardamonin analogs, DHC and DHMC, were found to decrease NF-κB activation and induce apoptosis in specific lung cancer cells [182]. Finally, cardamonin was reported to inhibit cell proliferation and induce cell cycle arrest in specific lung cancer cell lines while inhibiting p70S6K and mTOR activation [183].

### 8.8. Effects of Cardamom on Multiple Myeloma

Multiple myeloma is a type of blood cancer that affects plasma cells [184]. Studies investigating the potential therapeutic effects of cardamonin on multiple myeloma revealed that pretreatment with cardamonin led to increased caspase-3 and PARP activation, suppressing various anti-apoptotic proteins [185,186]. Cardamonin also diminished the activation of the NF-κB pathway by enhancing IKK and IkBA phosphorylation, and lowering the levels of ICAM-1, COX-2, and VEGF [185,186].

### 8.9. Effects of Cardamom on Ovarian Cancer

Ovarian cancer is a prevalent form of gynecological malignancy worldwide, and its five-year survival rate falls within the range of 20–47% [187]. Studies have shown that cardamonin exhibits anti-proliferative effects on SKOV3 and ovarian cancer cells by inducing apoptosis and reducing the levels of Bcl-2, survivin, and the mTOR cascade [100]. Additionally, cardamonin has been found to cause cell cycle arrest in the G2/M phase. Notably, research has demonstrated that the combined treatment of cardamonin with cisplatin yields a stronger and synergistic anti-cancer effect [69]. Another study focusing on SKOV3 ovarian cancer cells has revealed the anti-proliferative properties of cardamonin [188]. The compound also enhances autophagy by removing damaged cells by suppressing mTORC1 pathways and H2K expression [188].

### 8.10. Effects of Cardamom in Prostate Cancer

Prostate cancer affects one in every nine men worldwide, with older men being more at risk [189]. According to Zhang et al. (2017), cardamonin has been found to efficiently block the STAT3 signaling cascade, which can help repress the growth and viability of prostate cancer cells [101]. Additionally, Pascoal et al. (2018) demonstrated the effect of isolated chalcone on PC-3 cell lines, showing that cardamonin decreased the proliferation and viability of PC-3 cells through reduced expression of NF-κB [190].

## 9. Conclusions

A critical analysis of the existing literature on the medicinal properties and therapeutic uses of Elettaria cardamomum (cardamom) reveals that this spice not only enhances food flavor but also offers various health benefits. Research indicates that cardamom’s diverse bioactive compounds contribute to multiple health advantages. Multi-drug resistance and cancer present significant challenges to the medical field, yet bioactive compounds from natural spices, such as cardamom, provide hope in combating these issues. Cardamom demonstrates significant potential in fighting pathogenic bacteria, inhibiting tumor formation, curbing unwanted immune responses, and addressing other health concerns. These properties underscore the high interest in cardamom and its bioactive compounds.

In conclusion, it is evident from the current body of research that cardamom holds significant potential as a powerful anti-cancer agent. Many clinical studies used a daily intake of 3 g of cardamom, which exceeds typical dietary consumption and represents a pharmacological dose. A regular dietary intake of cardamom is generally around 0.5 to 1 g per day, whereas the 3-g dosage in the studies was intentionally chosen to explore potential therapeutic benefits beyond what is achievable through regular dietary intake. In many anti-cancer studies, cardamom extracts are often administered at doses ranging from 100 mg/kg to 200 mg/kg body weight in animal models, while clinical trials have used cardamom powder at up to 3 g daily for several weeks. Cardamom essential oil has also been tested in concentrations of 1% to 5% in various treatment regimens. It is important to consider that most of these anti-cancer studies were conducted in vitro, restricting their direct application in clinical settings. When cardamom is consumed through the diet, complex factors mediate its stability and utilization in cells, whereas, in in vitro studies, we see the direct effect of cardamom. Therefore more in vivo studies require to validate these effects and better understand cardamom’s potential as a cancer treatment. Nevertheless, cardamom’s diverse mechanisms of action justify the need for further exploration of its potential use in clinical settings.

## Figures and Tables

**Table 3:** Some important major bioactive compounds of *Elettaria cardamomum* and their targets.

Number	Name and Structure	Targets	Reference
1	Kaempferol 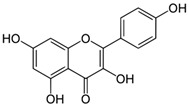	Increase the activity of PGC-1α and estrogen-related receptor α and inhibits cell proliferation and invasion in retinoblastoma via the Wnt/β-catenin signaling pathway	[56,57]
2	Luteolin 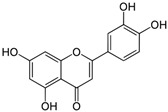	Phosphatidylinositol 3′-kinase (PI3K)/Akt, nuclear factor kappa B (NF-kappaB), and X-linked inhibitor of apoptosis protein (XIAP)	[58]
3	Pelargonidin 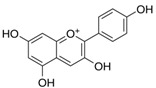	Inhibitors of NF-κB	[59]
4	Quercetin 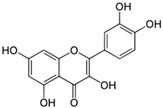	Quercetin inhibits lipopolysaccharide (LPS)-induced tumor necrosis factor α (TNF-α) production in macrophages	[60]
5	Resveratrol 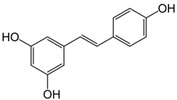	Inhibit ribonuclease reductase [1] or COX-2 activity [2]	[61,62]
6	Eucalyptol 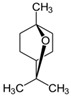	1,8-Cineol inhibits nuclear translocation of NF-κB p65 and NF-κB-dependent transcriptional activity	[63]
7	D-Limonene 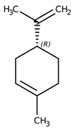	Inhibit PI3K/Akt/IKK-α/NF-κB p65 Signalling	[64]
8	Cymene	Modulate the calcium channel currents	[65]
9	α-Pinene	Suppress MAPKs and the NF-κB Pathways	[66]
10	Linalool	Modulate MAPK and NF-κB signaling	[67]
11	Borneol	Regulate HIF-1a expression via mTORC1/eIF4E pathway	[68]
12	Cardamonin	Target mTOR, NF-κB, Akt, STAT3, Wnt/β-catenin and COX-2	[14]
13	Indole-3-carbinol	Disrupt of NFκB nuclear localization and transcriptional activity, and induction of a G1 cell cycle arrest.	[69]
14	Diindolylmethane	Modulate NF-κB/Wnt/Akt/mTOR pathways	[70]

**Table 4:** Anti-cancer activity of *Elettaria cardamomum* against different types of cancer.

Type of Cancer	Tested Using	Reference
Breast cancers	Cardamonin and cardamom seed extract	[36,95,96,97]
Colon Carcinogenesis	Cardamom crude extract and cardamonin	[89,98]
Melanoma	Cardamonin	[99]
Ovarian cancers	Cardamonin	[100]
Prostate cancers	Cardamonin	[101]
Forestomach papillomagenesis	Cardamom crude extract	[91]
Pancreatic cancer	Kaempferol	[102]
Skin carcinogenesis	Cardamom powder	[90]
non-melanoma skin cancer	Cardamom crude extract	[103]
Head and Neck Squamous Cell Carcinoma	Cardamom oil	[104]
Bladder Cancer	Cardamomin	[105]

**Table 5:** Antioxidant activity of *Elettaria cardamomum* by different methods.

Measurement of Anti-Oxidative Property of Cardamom	Tested Using	Reference
2, 2,-diphenyl-1-picrylhydrazyl (DPPH) radical scavenging activity	Cardamom seed extract and total extract	[45,118]
NO radical scavenging assay	Cardamom crude extract	[122]
FRAP assay	Cardamom crude extract	[123]
Reducing power assay	Cardamom crude extract	[124]
ABTS Radical Cation Decolourisation Assay	Cardamom oil	[49]

**Table 6:** Anti-bacterial activity of *Elettaria cardamomum* against various bacteria.

Name of Bacteria	Reference
*Helicobacter pylori*	[133]
*Escherichia coli*, *Salmonella typhi*, *Bacillus cereus*, *Bacillus subtilis*, *Streptococcus pyogenes*, and *Staphylococcus aureus*	[134]
*Staphylococcus aureus* and *Proteus Mirabilis*	[135]
*Klebsiella pneumoniae*, *Pseudomonas aeruginosa*, *Salmonella typhi*, *Shigella dysenteriae*, *Shigella sonnei*, *Staphylococcus aureus*, *Streptococcus-β-haemolytica*, *Bacillus subtilis*, *B. megaterium*, and *Sarcina lutea*	[136]
*Staphylococcus aureus*, *Streptococcus pneumonia*, *S.epidermidis*, *P. aeroginosa*, *K. pneumonia*, *Proteus mirabilis*, *Enterobacter* spp. *Acinetobacter*, *E. coli*, *Serretia* spp. and *Salmonella typhi*	[131]
*E. coli*	[137]
*EPEC*, *L. monocytogenes*, *B. pumilus* and *E. coli*	[45]
*E. coli*, *S. typhi*, *S. pyogenes*, *S. aureus*, *B. subtilis* and *B. cereus*	[134]
